# High prevalence of polypharmacy and nervous system medications in people with HIV: a cross-sectional analysis

**DOI:** 10.1038/s41598-026-36832-4

**Published:** 2026-01-27

**Authors:** Aida López López, Alexandre Pérez González, Jacobo Alonso Domínguez, Antonio Ocampo, Celia Miralles, Luis Morano, José Aguayo Arjona, Noemí Martínez López de Castro, Eva Poveda

**Affiliations:** 1 Virology and Pathogenesis Research Group, Galicia Sur Health Research Institute (IIS Galicia Sur), Complexo Hospitalario Universitario de Vigo, SERGAS-UVIGO, 36213 Vigo, Spain; 2 Department of Pharmacy, Complexo Hospitalario Universitario de Vigo, Sergas, Vigo, Spain; 3 Infectious Diseases Unit, Department of Internal Medicine, Complexo Hospitalario Universitario de Vigo, Sergas, Vigo, Spain; 4 Infectious Diseases Unit, Complexo Hospitalario Universitario de Vigo, Sergas, Vigo, Spain; 5 Statistics and Methodology Unit, Galicia Sur Health Research Institute (IIS Galicia Sur), SERGAS-UVIGO, Vigo, Spain; 6 Innovation in Clinical Pharmacy (I-FARMA-Vigo) Research Group, Galicia Sur Health Research Institute (IIS Galicia Sur), SERGAS-UVIGO, Vigo, Spain

**Keywords:** HIV, Polypharmacy, Psychotropic drugs, Multimorbidity, Non-antiretroviral medication, Comorbidities, Infectious diseases, HIV infections, Viral infection

## Abstract

Polypharmacy is increasingly prevalent among people living with HIV (PLWH), especially as they age and manage multiple comorbidities. This cross-sectional study analyzed data from 268 PLWH in Vigo, Spain (2020–2023), revealing an aging cohort (mean age 49.8 years) and a 51.9% prevalence of multimorbidity. Descriptive, bivariate, and multivariable logistic regression analyses were performed. Polypharmacy, defined as the chronic use of ≥ 5 non-antiretroviral drugs, was observed in 35.7% of participants, increasing among older adults (≥ 50 years, 50.7%; *p* < 0.001) and those living with HIV for > 10 years (43.0%; *p* = 0.004). Nervous system medications (47.0%), alimentary tract/metabolism drugs (36.2%), and cardiovascular drugs (34.3%) were the most common. Psychotropic drugs were frequent, particularly anxiolytics (24.8%) and antidepressants (22.9%). In multivariable analysis, anxiolytic use was associated with older age (OR = 1.03; *p* = 0.038), female sex (OR = 1.97; *p* = 0.042), current smoking (OR = 3.74; *p* = 0.002), and past cocaine use (OR = 2.52; *p* = 0.008); antidepressant use with past (OR = 3.46; *p* = 0.015) and current smoking (OR = 4.46; *p* = 0.001). These findings highlight the complexity of managing polypharmacy in aging PLWH and underscore the need for strategies to optimize medication use.

## Introduction

The landscape of human immunodeficiency virus (HIV) treatment and demographics has changed significantly in recent years, driven by remarkable advancements in antiretroviral therapy (ART). These developments have greatly improved the life expectancy of people living with HIV (PLWH), bringing it closer to that of the general population^1^. This improvement is evidenced by the growing proportion of older adults (≥ 50 years) in this population. Globally, more than 4 million PLWH are aged 50 and older, and this number has been steadily increasing since 1995^2^. By 2030, modeling studies from high-income country cohorts predict that approximately 73% of PLWH will be older adults^3^.

However, inflammation persists in most PLWH, in spite of durable virological suppression and restored CD4 lymphocyte count. Alongside aging, various comorbidities associated with the state of chronic systemic inflammation caused by HIV infection itself may appear^4^. It is observed that PLWH over 50 years are more predisposed to develop diverse comorbidities compared to their seronegative peers. These comorbidities include non-AIDS-related neoplasms, diabetes mellitus, hypertension, or cardiovascular disease^5–8^. Moreover, mental health disorders such as major depression, generalized anxiety disorder, or substance use disorder are more frequent among PLWH^9–11^. Recent studies have revealed that neuroinflammation^12,13^ and stigma^10,14^ contribute to the high prevalence of depression among PLWH.

As the population ages and the burden of comorbidities rises, polypharmacy is expected to become more prevalent. Polypharmacy refers to the simultaneous use of multiple medications, typically defined as the regular use of five or more drugs. In the context of PLWH, this term specifically refers to the use of five or more non-antiretroviral (non-ART) medications^15^. Research suggests that the rate of polypharmacy among PLWH ranges from 15 to 51%^16–19^, a figure that is consistently higher across all age groups compared to the general population^20,21^. Population-based studies have highlighted which concomitant non- ART medication are most frequently used in PLWH, according to the Anatomical, Therapeutic, Chemical Classification System (ATC). The most frequent were nervous system medications regardless of age. This was followed by medications for the alimentary tract and metabolism, as well as cardiovascular medications, especially in adults over 50 years old^20,22,23^. When compared to the general population, it has been noted that some pharmacotherapeutic groups of medications are more likely to be used in PLWH. These include medications for acid-related disorders, β-blocking agents, antibacterials for systemic use, analgesics, antiepileptics, psycholeptics, psychoanaleptics, statins, medications for obstructive airway diseases, and antihistamines for systemic use^21–23^. Substance use is a clinically relevant factor that may influence comorbidity burden and pharmacological complexity; therefore, its assessment is essential to fully understand polypharmacy in PLWH^24^.

In this context, we conducted a cross-sectional study to explore the burden of polypharmacy in PLWH, and evaluate the pharmacotherapeutic profile of non-ART prescriptions, exploring the role of nervous system medications in an aging cohort of PLWH. Additionally, potential drug-drug interactions (DDIs) between ART and non-ART medications were identified.

## Methods

### Study design and population

This study employed a cross-sectional design to characterize the epidemiological, clinical, and pharmacological profiles of a cohort of PLWH in the Vigo health area from January 1, 2020, to December 31, 2023. At the time of conducting the study, this Infectious Disease cohort comprised 302 PLWH under clinical follow-up in the Infectious Disease Unit of Álvaro Cunqueiro Hospital in Vigo. Inclusion criteria for the study were as follows: participants had to have a confirmed diagnosis of HIV infection, be 18 years of age or older, and be enrolled in the Infectious Diseases cohort. Patients who did not attend the HIV unit between 2020 and 2023 were excluded from the study.

### Data collection and variables

Study data were collected from medical records and managed using REDCap (Research Electronic Data Capture) hosted at the Galicia Sur Health Research Institute Biobank (reference B.0000802). REDCap is a secure, web-based software platform designed to support data capture for research studies^25,26^. The database included comprehensive epidemiological, clinical and pharmacological data. Demographic information included sex, age, and origin. Substance use variables included smoking status and hazardous consumption of alcohol, cocaine, or heroin. Alcohol consumption was defined as hazardous for individuals consuming more than 17 units of alcohol per week for men and more than 11 units per week for women^27,28^. Data on methamphetamine use or chemsex practices were not collected in this study. Clinical variables included time living with HIV, modes of disease transmission, time on ART, latest CD4 + count, comorbidities, hospitalizations, and deaths during the study period. Only clinically relevant past hepatitis B virus (HBV) or hepatitis C virus (HCV) infections, as documented in the medical record, were included as comorbidities to better reflect the clinical complexity and therapeutic challenges in PLWH. Undetectable viral load was defined as a confirmed HIV RNA level below 50 copies/mL. Pharmacological variables included current ART regimen and co-medications, defined as non-ART chronic medication (taken for ≥ 6 months). Polypharmacy was defined as the simultaneous and chronic use of five or more non-ART drugs^15^. Herbal drugs and over-the-counter remedies were not considered, and combination medications containing two or more pharmacologically active agents were counted as a single drug. Potential DDIs between ART and non-ART medication were identified using the University of Liverpool (UoL) Drug Interactions Database. This tool offers a comprehensive, evidence-based classification of interactions relevant to PLWH. It uses a color-coded system where green indicates no significant interaction, amber signals a potential interaction requiring caution and monitoring, and red flags denote high-risk interactions. For this study, we focused on moderate (orange) and high-risk (red) interactions to capture clinically relevant drug combinations in our cohort^29^.

### Data analysis

For the descriptive study, values were expressed as absolute numbers and percentages, and as medians and interquartile ranges (IQRs). A bivariate analysis was conducted to explore associations between independent variables and each binary outcome of interest. Separate multivariable binary logistic regression models were performed for each outcome related to nervous system medication use (e.g., use of anxiolytics yes/no, use of antidepressants yes/no). All analyses were conducted using IBM SPSS Statistics for Windows. Statistical tests were two-sided, and a *p* value < 0.05 was considered statistically significant.

### Compliance with ethical standards

Patients in the Infectious Diseases cohort, assigned to the IISGS Biobank, signed an informed consent for the transfer of data. The informed consent was signed during the clinical follow-up of the patients. All collected data were pseudonymized, securely stored, and only accessible to authorized study personnel. The research project was approved by the Galician Committee on the Ethics of Research involving Medicinal Products (CEIm-G) under approval notice (Registration Code: 2024/019). All research was conducted in accordance with relevant guidelines and regulations, ensuring compliance with the Declaration of Helsinki for studies involving human participants.

## Results

### Clinical and epidemiological characteristics of the cohort

A total of 268 PLWH met the inclusion criteria and were included in the study, of whom 180 (70.1%) were cisgender men, 77 (28.7%) cisgender women and 3 (1.1%) transgender individuals. The average age was 49.8 years, ranging from 19 to 88 years, with 50.7% of participants being over 50 years old. The majority were of Spanish origin (84%), followed by Latin American (9.7%). Regarding substance use, 27.2% drank alcohol, 50.4% were active smokers, 5.5% were active cocaine users, and 1.1% were active heroin users (Table 1).

**Table 1:** Socio-demographics and substance use.

	Total (N = 268) n (%)
Age (years) (mean = 49.8, range 19–88)	
18–30	17 (6.3)
31–49	115 (42.9)
≥ 50	136 (50.7)
Sex	
Cisgender men	180 (70.1)
Cisgender women	77 (28.7)
Transgender	3 (1.1)
Origin	
Spanish	225 (84.0)
Latin America	26 (9.7)
African	7 (2.6)
Other	9 (3.4)
Unknown	1 (0.4)
Alcohol consumption*	
Current high-risk alcohol use	73 (27.2)
Former high-risk alcohol use	33 (12.3)
No high-risk alcohol use	194 (59.7)
Tobacco use	
Current smoker	135 (50.4)
Former smoker	37 (13.8)
Never smoker	95 (35.4)
Unknown	1 (0.4)
Heroin use	
Current heroin user	3 (1.1)
Previous heroin user	70 (26.1)
No heroin use	188 (72.4)
Cocaine use	
Current cocaine use	15 (5.6)
Previous cocaine use	69 (25.7)
No cocaine use	183 (68.3)

High-risk alcohol use: consuming more than 17 units per week for men and consuming more than 11 units per week for women.

The average time living with HIV among participants was 17.8 (± 9.84) years. The predominant modes of disease transmission categories were men who have sex with men (MSM) (35.8%) (Table 2). Almost all (98.1%) were on ART with undetectable viral load (91.7%). The median time on ART was 15 (± 8.96) years. The average latest CD4 + count was 746 (540–1029) cells/μl.

**Table 2:** Clinical characteristics of the study population.

	Total (N = 268) n (%)
Time living with HIV (years)	
< 10	56 (20.9)
10–19	71 (26.5)
> 20	94 (35.1)
Time on ART (years)	15.2 (± 8.96)
**ART**	
INSTI based regimen	224 (83.6)
BIC/FTC/TAF^a^	97 (36.2)
DTG/3TC^b^	85 (31.7)
DTG/RPV^c^	30 (11.2)
DTG/ABC/3TC^d^	8 (3.0)
Other INSTI based regimen	4 (1.5)
IP based regimen	13 (4.9)
DRV/c/FTC/TAF^e^	5 (1.9)
NRTI^f^ + NNRTI^g^ regimen	13 (4.9)
HIV transmission category	
Men who have sex with men	96 (35.8)
Heterosexual	89 (33.2)
Intravenous drug user	69 (25.7)
Blood product transfusion	5 (1.9)
Vertical transmission	6 (2.2)
Unknown	3 (1.1)
**Comorbidities**	
Arterial hypertension	41 (15.3)
Diabetes mellitus	17 (6.4)
Dyslipidemia	70 (26.1)
Bone disorders	
Osteopenia	79 (41.6)
Osteoporosis	31 (16.3)
Hepatitis B virus infection	
Chronic active HBV infection	3 (1.1)
Past HBV infection	91 (34.0)
Hepatitis C virus infection	
Chronic active HCV infection	3 (1.1)
Past HCV infection	84 (31.3)
Ischemic heart disease	9 (3.4)
Peripheral arterial disease	7 (2.6)
Cerebrovascular disease	8 (3.0)
Chronic obstructive pulmonary disease (COPD)	25 (9.3)
Active solid organ cancer	12 (4.5)
Mental health comorbidities	
Substance use disorders	41 (15.3)
Opioids	33 (12.3)
Cocaine	9 (3.4)
Alcohol	8 (3.0)
Hypnotic and sedatives	8 (1.1)
Cannabinoids	5 (1.9)
Other psychoactive substances	2 (0.7)
Harmful use of multiple drugs (no dependence detected)	2 (0.7)
Schizophrenia	5 (1.9)
Mood disorders (affective)	10 (3.7)
Bipolar disorder	2 (0.7)
Recurrent major depressive disorder	2 (0.7)
Symptoms of depressive disorder	6 (2.3)
Anxiety disorders	30 (11.2)
Mixed anxiety-depressive disorder	15 (5.6)
Generalized anxiety disorder	11 (4.1)
Severe stress reactions and adjustment disorders	9 (3.4)
Panic disorders	2 (0.7)
Other anxiety disorders	7 (2.6)
Sleep disorders	11 (4.1)
Alzheimer’s dementia	1 (0.4)
Personality disorders	3 (1.1)
Paraphilias	1 (0.4)
Attention deficit hyperactivity disorder	1 (0.4)

^a^BIC/FTC/TAF: bictegravir/emtricitabine/tenofovir alafenamide; ^b^DTG/3TC: dolutegravir/lamivudine; ^c^DTG/RPV: dolutegravir/rilpivirine; ^d^DTG/ABC/3TC: dolutegravir /abacavir/lamivudine; ^e^DRV/c/FTC/TAF: darunavir/cobicistat/emtricitabine/tenofovir alafenamide; ^f^NRTI: nucleoside reverse transcriptase inhibitor; ^g^NNRTI: non-nucleoside reverse transcriptase inhibitor.

The most common comorbidities included bone disorders (osteopenia and osteoporosis) (41.4%), past HBV infection (34.0%), past HCV infection (31.3%), and dyslipidemia (26.1%) (Table 2). All comorbidities were statistically more frequent among individuals over 50 years of age, except for COPD, which had similar rates between younger and older adults (9.8% vs. 8.8%; *p* = 0.773). All clinical characteristics were similar between men and women, except for a significantly higher prevalence of HBV past infection among men (40.4% vs 22.1%; *p* = 0.004). HBV and HCV infections were significantly more frequent among heroin (52.1% vs. 13.9%; *p* < 0.001, and 78.2% vs. 2.8%; *p* < 0.001, respectively) and cocaine users (45.7% vs. 24.3%; *p* < 0.001, and 59.8% vs. 18.3%; *p* < 0.001, respectively). More than half of the participants (139, 51.9%) had multimorbidity, defined as the presence of two or more chronic health conditions excluding HIV. Additionally, 79 (29.6%) participants were hospitalized at least once during the study period and 12 participants (4.5%) died. The causes of death were as follows: 7 (2.6%) neoplasms, 1 non-AIDS infectious disease (0.4%), 1 liver cirrhosis (0.4%), 1 neurological disorder (0.4%) and 1 suicide (0.4%). Among the neoplasms, anal cancer was the most frequent (2, 0.7%).

### Pharmacotherapeutic profile of the cohort across age groups

Polypharmacy was observed in 92 PLWH (35.7%). No differences were found in the rate of polypharmacy between men and women. We found a higher rate of polypharmacy among heroin users (49.3% vs. 29.9%; *p* = 0.003), adults over 50 years old (50.7% vs. 26.6%; *p *< 0.001) and those who had been living with HIV for more than 10 years (43.0% vs. 14.5%; *p* = 0.004). Additionally, the mean age of individuals with polypharmacy was significantly higher compared to those without (57.2 vs. 45.8 years; *p* < 0.001). Almost all participants (254, 94.8%) were prescribed co-medications, with 140 (50.2%) taking three or more co-medications. The degree of polypharmacy was moderate (5–9 drugs) in 68 PLWH (26.4%) and severe (> 10 drugs) in 19 PLWH (7.4%). A significantly higher proportion of polymedicated participants required hospital admission during the study period compared to those who did not (57.0% vs. 26.1%; *p* < 0.001).

The most common ART regimen was based on integrase strand transfer inhibitors (INSTIs), prescribed to 224 participants (83.6%). The most frequently used combinations were bictegravir, emtricitabine and tenofovir alafenamide (BIC/FTC/TAF) (97, 36.2%), dolutegravir plus lamivudine (DTG/3TC) (85, 31.7%), and dolutegravir with rilpivirine (DTG/RPV) (30, 11.2%). This was followed by protease inhibitors regimen (PIs) (16, 6.0%), a combination of INSTIs and PIs (13, 4.9%), and regimen based on nucleoside reverse-transcriptase inhibitors (NRTIs) combined with non-nucleoside reverse-transcriptase inhibitors (NNRTIs) (13, 4.9%) (Table 2).

The most prescribed non-ART medications in our cohort belonged to the following main anatomical groups: nervous system (N) (126, 47.0%), alimentary tract and metabolism (A) (97, 36.2%), cardiovascular system (C) (92, 34.3%), and respiratory system (R) (40, 14.9%). When stratified by age, we observed that older adults (≥ 50 years) had a significantly higher proportion of medication use in several anatomical groups compared to younger individuals (< 50 years), including alimentary tract and metabolism (A) (53.7% vs. 18.2%; *p* < 0.001), blood and blood forming organs (B) (16.9% vs. 4.5%; *p* = 0.001), cardiovascular system (C) (55.9% vs. 12.1%; *p* < 0.001), musculoskeletal system (M) (19.9% vs. 3.8%; *p* < 0.001), and notably, the nervous system (N) (58.1% vs. 35.6%; *p* < 0.001). Regarding specific pharmacotherapeutic classes, the most frequently prescribed drugs overall were anxiolytics (68, 25.4%), antihypertensives (including diuretics, beta-blockers, calcium channel blockers, and agents acting on the renin-angiotensin system) (67, 25.0%), hypolipidemic agents (67, 25.0%), antidepressants (62, 23.1%), vitamin D and analogues (57, 21.3%), and proton pump inhibitors (42, 15.7%). Importantly, the use of anxiolytics was significantly higher in older adults compared to younger individuals (32.4% vs. 18.2%; *p* = 0.006). Similarly, other drug classes such as antihypertensives, lipid modifying agents, vitamin D and analogues, proton pump inhibitors, and analgesics were also significantly more prevalent in the older age group. In contrast, no significant age-related differences were observed in the use of antidepressants, hypnotics and sedatives, or drugs for obstructive airway diseases (Table 3).

**Table 3:** Most common pharmacotherapeutic groups used, by age. Significant values are in [bold].

	Total (N = 268)		< 50 years (n = 132)		≥ 50 years (n = 136)		OR	95% CI	***p***
	n	%	n	%	n	%			
Anxiolytics	68	25.4	24	18.2	44	32.4	2.15	1.22–3.81	**0.006**
Antihypertensives^a^	67	25.0	5	4.3	62	45.6	21.28	8.29–54.62	** < 0.001**
Lipid modifying agents	67	25.0	12	9.1	55	40.4	6.79	3.42–13.47	** < 0.001**
Antidepressants	62	23.1	29	22.0	33	24.3	1.14	0.64–2.01	0.667
Vitamin D and analogues	57	21.3	14	10.6	43	31.6	3.90	2.01–7.55	** < 0.001**
Proton pump inhibitors	42	15.7	6	4.5	36	26.5	7.56	3.01–18.66	** < 0.001**
Analgesics	41	15.3	11	8.3	30	22.1	3.11	1.49–6.52	**0.002**
Hypnotics and sedatives	25	9.3	11	8.3	14	10.3	1.27	0.55–2.89	0.676
Drugs for obstructive airway diseases	32	11.9	12	9.1	20	14.7	1.72	0.81–3.69	0.188

^a^Antihypertensives: including diuretics, beta-blockers, calcium channel blockers, and agents acting on the renin-angiotensin system.

### Nervous system medication profile

Our findings revealed that medications targeting the nervous system were the most frequently used among PLWH (Table 4). Psycholeptics were the most prevalently prescribed in 31.3% of participants. This category includes anxiolytics, hypnotics/sedatives, and antipsychotics. Anxiolytics were prescribed to 25.4% of participants, all of which were benzodiazepine derivatives. Intermediate-acting benzodiazepines were the most commonly used (56, 82.3%), of which alprazolam (25, 44.6%) and lorazepam (22, 39.2%) were the most prevalent. Hypnotics and sedatives were used by 8.4% of participants, with benzodiazepine derivatives being the most common (4.9%), such as lormetazepam (3.4%). Antipsychotics were prescribed to 8.0% of the participants.

**Table 4:** Nervous system medications among the study population.

Therapeutic and pharmacological subgroups	Total (N = 268) n (%)	Most prescribed drugs
N02. Analgesics	39 (14.9)	
N02A. Opioids	17 (6.5)	Tramadol, fentanyl, morphine
N02B. Other analgesics and antipyretics	26 (9.9)	Paracetamol, pregabaline, metamizole
N02C. Antimigraine	2 (0.8)	Zolmitriptan, sumatriptan
N03. Antiepileptics	15 (5.7)	
N03A. Antiepileptics	15 (5.7)	Clonazepam, levetiracetam, lamotrigine
N04. Antiparkinsonians	1 (0.4)	
N04A. Anticholinergic agents	1 (0.4)	Biperidene
N05. Psycoleptics	82 (31.3)	
N05A. Antipsychotics	21 (8.0)	Olanzapine, quetiapine, risperidone
N05B. Anxiolytics	65 (24.8)	Alprazolam, lorazepam, diazepam
N05C. Hypnotics and sedatives	22 (8.4)	Lormetazepam, zolpidem, clomethiazole
N06. Psychoanaleptics	61 (23.3)	
N06A. Antidepressants	60 (22.9)	Escitalopram, mirtazapine, sertraline
N06B. Psychostimulant agents for attention deficit disorder	3 (1.1)	Citicoline
N07. Other pharmacological agents on the nervous system	22 (8.4)	
N07B. Drugs used in addictive disorders	21 (8.0)	Methadone, disulfiram, buprenorphine- naloxone
N07C. Antivertigo preparations	1 (0.4)	Betahistine

Therapeutic and pharmacological subgroups are not mutually exclusive.

Psychoanaleptics were the next most prevalent (23.3%), with antidepressants being the most common (22.9%). The predominant group was selective serotonin reuptake inhibitors (9.5%), particularly escitalopram (3.8%), followed by serotonin–norepinephrine reuptake inhibitors (6.1%).

Analgesics were used by 14.9% of participants, with opioids accounting for 6.5% and other analgesics and antipyretics for 9.9%, including paracetamol, pregabalin, and metamizole. Other pharmacological agents affecting the nervous system were prescribed to 8.4% of participants, mainly for opioid dependence (8.0%) and represented by methadone (7.3%).

### Factors associated with the use of nervous system medication

Significant differences in the use of nervous system medications were observed according to age, sex, ART regimen, and substance use. Multivariable logistic regression models were used to identify independent factors associated with the use of anxiolytics, hypnotics and sedatives, antipsychotics, antidepressants, and analgesics.

Anxiolytic use was associated with older age, female sex, active smoking, and past cocaine use. Hypnotic and sedative use was linked to PI-based ART. Antipsychotic use showed strong associations with both past and current cocaine use. Among psychoanaleptics, antidepressant use was related to both current and past smoking, while analgesic use was associated with older age. The specific variables included in each model, along with their corresponding odds ratios, 95% confidence intervals, *p* values, and area under the curve (AUC), are presented in Table 5.

**Table 5:** Multivariable binary logistic regression models for predictors of nervous system medication use. Significant values are in [bold].

Nervous system medication	Independent variables	OR	IC 95%	*p*	AUC
Anxiolytic	Age	1.03	1.02–1.06	**0.038**	0.753
	Female sex	1.97	1.03–3.77	**0.042**	
	Past smoking	2.48	0.87–7.10	0.091	
	Current smoking	3.74	1.64–8.54	**0.002**	
	Past cocaine use	2.52	1.27–4.99	**0.008**	
	Current cocaine use	3.00	0.93–9.63	0.065	
	ART^a^ regimen: PI^b^ + INSTI^c^	0.19	0.22–1.59	0.124	
Hypnotic and sedative	ART regimen: PI	3.78	1.12–12.82	**0.033**	0.555
Antipsychotic	Past cocaine use	3.77	1.35–10.57	**0.012**	0.726
	Current cocaine use	16.48	4.58–59.25	** < 0.001**	
Antidepressant	Past smoking	3.46	1.27–9.34	**0.015**	0.645
	Current smoking	4.46	2.05–9.70	**0.001**	
Analgesic	Age	1.07	1.03–1.10	**0.001**	0.692

^a^ART: antiretroviral treatment; ^b^PI: protease inhibitors; ^c^INSTI: integrase strand transfer inhibitors.

### Drug-drug interactions

Overall, we detected that 32 (11.9%) PLWH experienced at least one DDI with ART: 25 (9.3%) had one, 6 (2.2%) had two, and 1 (0.4%) had three, with a total of 40 DDIs. Potential DDIs between ART and non-ART medications were identified using the UoL Drug Interactions Database. Among these interactions, 35 were classified as moderate risk (orange flag) and 3 as high risk (red flag). The therapeutic classes most frequently involved were antidepressants (6, 2.3%), lipid-lowering agents (6, 2.3%), antidiabetic medications (5, 1.9%), antianemic agents (4, 1.5%) and mineral supplements (4, 1.5%). The most commonly implicated drugs were atorvastatin (5, 1.9%) and metformin (5, 1.9%).

## Discussion

In this cohort of 268 PLWH, a notable proportion of participants were over 50 years old, reflecting the aging trend within the population. This aging is accompanied by an important burden of comorbidities and polypharmacy. Across the full study population, psychotropic drug use was highly prevalent despite the low reported rates of mental health disorders. Additionally, a history of substance use was prevalent among participants and may contribute to the complexity of care needs in this population.

These data on the aging HIV population and the increasing burden of comorbidities are consistent with predictive modeling by Smit et al., based on the Dutch ATHENA cohort^30^. However, the burden of polypharmacy has already surpassed the model’s 2030 predictions. Nearly twice as many participants are taking at least one co-medication (94.8% vs. 54%), and 2.5 times as many participants are taking three or more medications (50.2% vs. 20%)^30^. In line with these data, we found that the rate of polypharmacy was higher (35.7%) in our cohort than in individuals without HIV (22.1%)^20^, and falls within the range observed in other studies (8.9–51%)^16–20^. It is important to note that, in our study, polypharmacy was defined as the concurrent use of five or more chronic non-ART medications. The variability in reported prevalence across studies is largely explained by differences in how polypharmacy is defined. For instance, Gimeno-Gracia et al. applied a stricter definition, requiring a cumulative defined daily dose (DDD) > 180 per drug, which led to much lower prevalence estimates (8.9% in men, 11.3% in women)^22^. Conversely, Psomas et al. reported a prevalence of 51% by including herbal products, over-the-counter medications, and counting each active compound in fixed-dose combinations separately^18^.

When examining medication use by ATC classification, nervous system drugs emerged as the most frequently used overall, with a notably higher prevalence among participants aged 50 and older. Cardiovascular medications were the second most common in this older group, reflecting the increased burden of related comorbidities. In contrast, younger adults showed a relatively higher use of medications for the alimentary tract and metabolism. These findings agree with other population-based studies^20,22^, except for anti-infective medications. This discrepancy arises because we only considered non-ART medications taken chronically (for ≥ 6 months), resulting in a lower observed proportion of anti-infectives for systemic use. Stratifying by age, we observed that the proportion of medications used in our cohort was generally higher among older adults. Individuals with polypharmacy had a significantly higher mean age compared to those without, further supporting the association between aging and increased treatment complexity. However, when analyzed by pharmacotherapeutic group, this trend persists except for medications used for agents against respiratory tract disorders, antidepressants, and hypnotics and sedatives, for which no significant differences were found between younger and older adults. The high use of agents against respiratory tract disorders could be explained by the fact that we found no differences in COPD between those over and under 50 years of age.

Compared with the general population31, antidepressant and hypnotic/sedative use in our cohort showed a distinct profile. While in the general population these medications increase with age and are more prevalent among women, such patterns were not observed in our cohort, suggesting that HIV may influence psychotropic drug use independently of demographic characteristics. Supporting this, a Danish population-based study reported higher use of antidepressants, anxiolytics, hypnotics, and antipsychotics among PLWH compared to the general population, even after excluding individuals with a history of injecting drug use or HCV infection32. In that cohort, increased use was associated with older age and longer time since HIV diagnosis, but not with ART exposure, reinforcing the idea that HIV infection itself and its clinical context may modify psychotropic drug prescribing patterns.

Our cohort exhibited a distinct psychotropic drug profile compared to the general population^31^. While national primary care data show that antidepressant and sedative use typically increases with age and is more frequent in women, these patterns were not observed in our study, suggesting that HIV may modify psychotropic drug consumption independently of traditional demographic factors. These observations align with a Danish population-based study, which also reported higher use of antidepressants, anxiolytics, hypnotics, and antipsychotics among PLWH compared to the general population, even after excluding individuals with a history of injecting drug use or HCV co-infection^32^. In that cohort, increased use was associated with older age and longer time since HIV diagnosis, but not with ART exposure. Together, these findings reinforce the notion that HIV infection and its clinical context are important determinants of psychotropic medication patterns.

Regarding antidepressants, the prevalence in our cohort (22.9%) is comparable to the rate observed by Cholera et al. (23–24%)^33^. Conversely, the use of anxiolytics in our cohort aligns with trends observed in the general population^31^, with usage increasing with age and being more common among women. Notably, female participants in our cohort consumed more anxiolytics than their male counterparts, although there were no significant differences in the consumption of other nervous system medications. This underscores gender differences in mental health, with older women being more likely than men to experience common mental disorders such as depression and anxiety^34^. The high prescription rate of psychotropic medications in the cohort contrasts with the low prevalence of documented psychiatric comorbidities. This prevalence is lower than that reported in other studies^8,11,35–37^, suggesting potential under-reporting or under-diagnosis of these conditions. This is consistent with previous studies highlighting the difficulties in diagnosing neuropsychiatric comorbidities, such as depression and anxiety, in PLWH^10,38,39^. A study from the University of Pennsylvania using electronic health records from the Center for AIDS Research Longitudinal Database found that, although major depressive disorder was adequately documented, other conditions (including post-traumatic stress disorder, substance or alcohol use disorders, and sleep or adjustment disorders) were significantly under-registered compared to national estimates for this population^40^. Similarly, a recent study conducted in Spain among HIV specialist physicians revealed that, despite acknowledging the importance of neuropsychiatric comorbidities, most clinicians reported limited training, low adherence to diagnostic guidelines, and infrequent use of screening tools. These factors likely contribute to the underdiagnosis and underdocumentation of such conditions^39^. Together, these findings suggest a systematic bias in clinical detection and reporting practices, potentially obscuring the true burden of psychiatric comorbidities in PLWH. The psychological impact of depressed mood, driven by HIV-related stress and reduced autonomy, as described by Ballester-Arnal et al.^41^, further underscores the importance of mental health care in PLWH and the need for tailored psychological support. The consensus paper on the clinical management of neuropsychiatric and cognitive comorbidity associated with HIV-1 infection highlights early detection, integrated care, and individualized interventions as key strategies^42^, which could reduce reliance on pharmacological treatment alone, especially in populations with high comorbidity burdens.

The use of non-ART medications appears to be influenced by substance use. High rates of smoking and alcohol consumption were observed, aligning with previous studies showing increased hazardous drinking, smoking, and illicit drug use in PLWH compared to the general population^9,43^. While current cocaine and heroin use rates are low compared to other studies in PLWH^9,44,45^, past use remains high. Our cohort analysis revealed that PLWH who are current or former users of tobacco or cocaine are at an increased risk of psychotropic drug use. Notably, active smoking and past cocaine abuse were linked to higher use of anxiolytics, suggesting a significant reliance on these medications among substance users. Antidepressant use was also more frequent among both current and former smokers. While a relationship between smoking and depression or anxiety has been established, the causal direction of this association remains unclear^46^. In a study by Fuster-Ruiz de Apocada et al., which aimed to determine the prevalence of drug use among PLWH in Spain, it was found that PLWH who used illicit drugs had a higher prevalence of consuming antidepressants, sedatives, hypnotics, opioids, methadone and erectile dysfunction medications^9^. Moreover, substance use adds complexity to the management of comorbidities and polypharmacy in PLWH, increasing the likelihood of DDIs and challenges with treatment adherence, which may contribute to poorer clinical outcomes in this population^24^. On the other hand, antipsychotic use in our cohort was also associated with active and past cocaine use. Cocaine use is known to be linked to mental health disorders, including psychosis, either directly due to the drug’s effects or indirectly through its role in exacerbating underlying psychiatric conditions^47,48^. These data indicate a strong intersection between substance use and nervous system medications. It emphasizes the need for comprehensive and integrated treatment approaches that address both substance use disorders and neuropsychiatric conditions.

Regarding the ART regimen, no significant differences were observed in the prevalence of mental health disorders across the different ART groups. However, certain antiretroviral drugs are more commonly associated with central nervous system adverse events, particularly INSTIs^49–52^. Neuropsychiatric adverse events associated with INSTIs are increasingly recognized in clinical settings, especially with dolutegravir. These adverse events include insomnia, dizziness, anxiety, depression, headaches, and cognitive impairments such as poor concentration and slow thinking, with reported discontinuation rates due to neuropsychiatric adverse events being notably higher than those observed in randomized clinical trials^50,51^. Certain populations, including elderly patients, women, and those initiating abacavir therapy concurrently with DTG, appear to be more susceptible to these adverse effects^50–52^. Newer PIs like darunavir tend to have a more favorable profile concerning central nervous system side effects^49^. Despite the lower risk of direct neuropsychiatric adverse events with PI-based regimens, we observed that the use of hypnotics and sedatives was independently associated with PI-based therapy. However, this association should be interpreted with caution, as the discriminatory capacity of the regression model was limited (AUC = 0.555), suggesting a weak ability to distinguish between users and non-users of hypnotics based on the variables included. Notably, insomnia and abnormal dreams have been documented as relatively common side effects of PI-based regimens^53,54^, which may contribute to the need for sleep-inducing medications in this patient population. Consequently, these results emphasize the need for comprehensive central nervous system monitoring, even in patients on regimens with a perceived lower risk of neuropsychiatric events.

The study revealed that 11.9% of PLWH in our cohort experienced at least one DDI, a prevalence lower than that observed in other population-based studies, which range from 17.2 to 35.0%^20,55,56^. This discrepancy may be explained by our focus on chronic medications, which could underrepresent interactions involving short-term or acute therapies. Although most interactions were not life-threatening, they required clinical attention to prevent adverse effects or reduced ART efficacy. Special focus is needed on interactions involving antidepressants, lipid-lowering agents, and antidiabetics. Proactive management, regular review, and optimization of treatment plans are essential to maintain ART effectiveness and minimize the risk of adverse drug reactions.

This study has several limitations. Its cross-sectional design precludes establishing causal relationships. Additionally, several potentially relevant variables, such as educational level, unemployment, and non-disclosure of HIV status, were not captured, which could influence the prevalence of depression, anxiety, and psychotropic medication use. Although our analysis included age stratification, the inclusion of a general adult population (≥ 18 years) may limit the specificity of conclusions regarding aging-related trends. Future studies focusing exclusively on older cohorts of PLWH are warranted. The relatively small sample size and the lower proportion of MSM compared to other PLWH cohorts may also limit the generalizability of our findings. Furthermore, data on reasons for hospital admission, quality of life, and methamphetamine or chemsex practices were not available, potentially leading to underestimation of behavioral risk factors, particularly among MSM, for psychotropic medication use. The number of transgender participants was also too small to allow meaningful subgroup analysis. An additional limitation is the potential underreporting of psychiatric diagnoses, as some may not have been captured in the hospital’s electronic health records, particularly those made outside the national health system, despite the high prevalence of psychotropic medication use.

In contrast, this study has several strengths, including comprehensive data collection on demographics, clinical factors, and pharmacology, allowing for an in-depth analysis of polypharmacy trends. By focusing on non-ART medications, it offers a broader view of medication use among PLWH, particularly in relation to aging and multimorbidity. The high prevalence of nervous system medications suggests potentially underdiagnosed mental health disorders in PLWH. This underscores the importance of addressing mental health in HIV management, a critical yet often underemphasized aspect of care.

In conclusion, this study highlights the substantial burden of polypharmacy among PLWH, a challenge that is intensifying with the aging population and rising rates of multimorbidity. The strong associations between polypharmacy, older age, longer time living with HIV and higher hospitalization rates emphasize the need for careful management and close monitoring of these patients. The high prevalence of non-ART medications, particularly those affecting the nervous system, further illustrates the complexity of care required for PLWH. This issue is exacerbated by substance use. Moreover, the study highlights the impact of demographic factors as age or sex on medication use, reflecting broader concerns about an aging population and gender disparities in mental health. The higher usage patterns of antidepressants, hypnotics, and sedatives compared to the general population indicate that HIV might influence the epidemiology of related mental health conditions. Targeted interventions are crucial to managing polypharmacy and optimizing pharmacotherapy, particularly concerning mental health and substance use disorders. Future research should prioritize improving the diagnosis and treatment of psychiatric conditions and exploring the impact of polypharmacy on health outcomes among PLWH. These efforts are crucial for enhancing the quality of life and clinical outcomes as PLWH continue to age. The complexity of care, driven by aging, polypharmacy, and multimorbidity, underscores the growing necessity for a holistic approach to HIV management.

## Data Availability

The datasets generated and analyzed during the current study are not publicly available, but are available from the corresponding author on reasonable request. Access will require adherence to appropriate ethical guidelines and approval from the relevant institutional review board.
